# The Diagnosis and Treatment of Diphtheria

**Published:** 1897-11-27

**Authors:** 


### THE DIAGNOSIS AND TREATMENT OF DIPHTHERIA.

It is satisfactory to find that several of the metropolitan sanitary authorities have now made arrangements for placing at the disposal of medical men practising in their districts an opportunity of obtaining a bacteriological examination and report in doubtful or suspected cases of diphtheria. Among the medical officers who now have in hand this additional means of combating this serious disease we may mention Dr. Sykes, of St. Pancras; Dr. Allan, of the Strand; and Dr. Wynter Blyth, of Marylebone. Dr. Bond, of Holborn, also has recommended its adoption in his district.

The circular letter, by Dr. Allan, which accompanies the sterilised swabs for the collection of the specimens, states that a report will be furnished, at latest, the day after the tube is received back at the laboratory (Sundays and holidays excepted), and it is urged that, as the diphtheria organism may persist in the throat for considerable periods after the cessation of clinical symptoms, one or more subsequent reports should be obtained, with a view to a continuance of local treatment, and isolation so long as the case remains infective.

Dr. Allan also says that on receipt of a written request he will arrange for the disinfection of articles of clothing (either belonging to the medical man, or to nurses or persons attending on the patients), which may have been exposed to infection. It is satisfactory to note that the Board is also prepared to disinfect rooms which have been occupied by patients suffering from measles, whooping cough, and tuberculous disease. This is a move quite in the right direction.

A recent resolution of the Metropolitan Asylums Board puts another very important means at our disposal for the management of this disease. At the meeting held last Saturday, Mr. R. M. Hensley presented a report from the General Purposes Committee recommending "That the present practice of supplying anti-toxic serum for patients suffering from diphtheria who are unable to obtain admission into the Board's hospitals be amended by placing in the hands of the several metropolitan medical officers of health, as well as in the hands of the Board's medical superintendents, a supply of anti-toxin for distribution to any general practitioner signing the necessary warrant." The recommendation was agreed to.

For some time back it has been the practice, when cases were refused admission by reason of there being no room in the hospitals, to send to the practitioner in attendance a warrant for a supply of anti-toxin. This anti-toxin, however, was only to be obtained at the offices of the Board or the laboratory on the Embankment, or at one or other of the Board's hospitals, and, moreover, the use of it was strictly limited to the cases which had been refused admission. This was obviously absurd, and savoured more of an attempt to protect the Managers from blame than of any serious desire to stem the current of the epidemic.

The new arrangement will, we hope, be found to work much more satisfactorily, and in view of the admirable reports on the use of anti-toxin by the medical officers of the Board's hospitals during the past two years (issued by the Metropolitan Asylums Board), we cannot too strongly urge the *early* use of this valuable remedy. The reports from the Northern Hospital as to the benefit to be derived from anti-toxin are by far the most striking, and there, of course (it being a hospital for convalescents from scarlet fever), the cases were picked out and treated as soon as ever they occurred.

At the same meeting it was resolved to enter into an arrangement with the Laboratories Committee of the Royal College of Physicians and the Royal College of Surgeons, for a supply of anti-toxin during the next seven years.

The expense involved in these schemes may possibly be considerable, but the loss of life which is now going on from diphtheria is so serious—good lives, too, of strong healthy children who are cut down by the disease in a few days—that no expense which is likely to be incurred in this way is worthy of being considered for a moment.

